# Induction of trained immunity in broiler chickens following delivery of oligodeoxynucleotide containing CpG motifs to protect against *Escherichia coli* septicemia

**DOI:** 10.1038/s41598-024-69781-x

**Published:** 2024-08-14

**Authors:** Iresha Subhasinghe, Khawaja Ashfaque Ahmed, Lisanework E. Ayalew, Hemlata Gautam, Shelly Popowich, Ayumi Matsuyama-Kato, Betty Chow-Lockerbie, Suresh K. Tikoo, Philip Griebel, Susantha Gomis

**Affiliations:** 1 Department of Veterinary Pathology, Western College of Veterinary Medicine, University of Saskatchewan, 52 Campus Drive, Saskatoon, SK S7N 5B4 Canada; 2 Atlantic Veterinary College, University of Prince Edward Island, 550 University Ave, Charlottetown, PE C1A 4P3 Canada; 3 Vaccinology and Immunotherapy, School of Public Health, University of Saskatchewan, 5D40 Health Sciences, 107 Wiggins Road, Saskatoon, SK S7N 5E5 Canada; 4 VIDO-InterVac, University of Saskatchewan, Saskatoon, SK S7N 5E3 Canada

**Keywords:** CpG-ODN, Trained immunity, Innate immune memory, Mitochondrial OXPHOS, Cellular glycolysis, Broiler chicken, Genetic engineering, Microbiology, Bacteria

## Abstract

Oligodeoxynucleotides containing CpG motifs (CpG-ODN) can promote antimicrobial immunity in chickens by enriching immune compartments and activating immune cells. Innate memory, or trained immunity, has been demonstrated in humans and mice, featuring the absence of specificity to the initial stimulus and subsequently cross-protection against pathogens. We hypothesize that CpG-ODN can induce trained immunity in chickens. We delivered single or multiple administrations of CpG-ODN to birds and mitochondrial oxidative phosphorylation (OXPHOS) and glycolysis of peripheral blood mononuclear cells were quantified using Seahorse XFp. Next, chickens were administered with CpG-ODN twice at 1 and 4 day of age and challenged with *Escherichia coli* at 27 days of age. The CpG-ODN administered groups had significantly higher mitochondrial OXPHOS until 21 days of age while cellular glycolysis gradually declined by 14 days of age. The group administered with CpG-ODN twice at 1 and 4 days of age had significantly higher survival, lower clinical score and bacterial load following challenge with *E. coli* at 27 d of age. This study demonstrated the induction of trained immunity in broiler chickens following administration of CpG-ODN twice during the first 4 days of age to protect birds against *E. coli* septicemia at 27 days of age.

## Introduction

One of the major problems in the poultry industry are economic losses associated with bacterial infections, particularly *Escherichia coli* (*E. coli*) and *Salmonella* species in neonatal chickens. In addition, *Salmonella* species associated with food-borne illnesses in humans commonly originate from contaminated poultry products^1,2^. Therefore, antimicrobial use (AMU) is important in the poultry industry to maintain the health and welfare of birds and to ensure safe poultry products for consumers. However, there are growing public concerns about the emergence of antimicrobial-resistant (AMR) strains of bacteria that will eventually adversely affect animal and human health and the environment. The poultry industry in many countries is transitioning into antimicrobial-free farming, but innovative alternative strategies to replace antibiotics such as novel vaccines and vaccine delivery systems are needed^3,4^.

Several alternatives, such as probiotics, are being used as substitutes for antibiotics. Previous studies indicate that probiotics, and prebiotics significantly improved feed conversion ratio and reduced stress as growth promoters but did not provide significant protection against bacterial challenge^5,6^. Oligodeoxynucleotides containing CpG motifs (CpG-ODN) is an immune stimulatory synthetic counterpart of bacterial DNA that acts as pathogen-associated molecular patterns (PAMP) in the vertebrate host that trigger a danger signal when the host encounters a pathogen^7^. In the past, we have demonstrated the immunoprotective properties of CpG-ODN in broiler chickens as a standalone antimicrobial agent against *E. coli* and *Salmonella* Typhimurium (*S*. Typhimurium) infections^8–14^. This immune modulation occurs through the activation of innate immunity directed through toll-like receptors (TLRs). CpG-ODN binds with TLR-21 in chickens which is a homologue of mammalian TLR-9^15,16^. We established that administration of CpG-ODN in broiler chickens enriches major immune compartments such as the spleen and lungs by increasing the recruitment of immune cells and inducing pro-inflammatory cytokines such as interferon-gamma (IFN-γ), interleukin (IL)-1β, IL-6, and IL-8, anti-inflammatory cytokines such as IL-10 and IL-4^14,17^.

Microbial invasion of the host is followed by a series of events leading to microbial killing mechanisms such as phagocytosis and intracellular microbial killing associated with oxygen-mediated or nitric oxide-mediated microbicidal activities^16,18^. Upon CpG-ODN binding and internalization, intracellular signaling pathways lead to the production of pro-inflammatory cytokines which triggers the activation of microbial killing mechanisms^19^. Moreover, during an immune response, profound metabolic changes are undergone to facilitate the signaling process and fulfill the energy demands of immune cells^20,21^. We recently provided evidence that CpG-ODN can increase serum metabolites including amino acids, choline, purines, betaine, and glucose allowing the host to increase the uptake of nutrients to enhance energy production and to assist immune cell proliferation and expression of receptors without favoring microbial proliferation^22^.

Metabolic reprogramming instigates specific cell functions of peripheral blood mononuclear cells (PBMCs)^23^. There are distinct metabolic pathways in immune cells to satisfy energy demands to accomplish and regulate cellular functions. Cellular glycolysis and mitochondrial oxidative phosphorylation (OXPHOS) are leading mechanisms to acquire energy demand^24–26^. Several recent studies in humans and mice have suggested that energy metabolism significantly regulates immune cell fate and functions by metabolic alterations of immune cells following exposure to pathogens^27^. Metabolites act as signaling molecules, cofactors, and substrates, impacting the activity of different enzymes involved in chromatin modification. This close interplay between metabolism and epigenetic reprogramming urges further exploration to understand the mechanisms underlying the different cellular metabolic pathways implicated in innate immune memory or trained immunity^28^.

Host cells can undergo epigenetic changes in response to various triggers. Cells that have undergone training, exhibit constant chromatin modifications which can impact the transcription machinery and ultimately lead to a sustained increase in gene transcription upon exposure to a secondary trigger. Trained immunity is based on non-permanent genetic changes that may occur in response to certain stimuli.^29,30^. Innate immunological memory or trained immunity featuring the absence of specificity to the initial stimulus, therefore, can grant cross-protection against subsequent bacterial or viral infections^31^. There is compelling evidence regarding trained immunity in mice^32–34^ and humans^35,36^. A recent study in mice demonstrated protection against pulmonary tuberculosis, *Candida albicans*, and *Schistosoma mansoni* infections following Bacillus Calmette-Guérin (BCG) vaccination^37^. The benefit of repeated administration of CpG-ODN has been shown in other species. For instance, increased protection was observed in mice against *Francisella tularensis* and *Listeria monocytogenes* challenge up to 2 weeks following repeated (2–4 times/month) intramuscular (IM) CpG-ODN administration^38^. In another study, two consecutive intravenous (IV) administrations of CpG-ODN (20 μg) had significantly increased protection for up to 2 weeks against *Staphylococcus aureus* mice for up to 2 weeks^39^. Nevertheless, CpG-ODN mediated trained immunity has not been investigated in chickens to date. In this study, we hypothesized that multiple exposures of broiler chickens to CpG-ODN can initiate trained immunity against bacterial infections. Therefore, the objectives of this study were to explore the ability of CpG-ODN to induce trained immunity in broiler chickens including the kinetics of energy production of immune cells in real time.

## Results

### The experimental design and timelines

**Figure 1:**
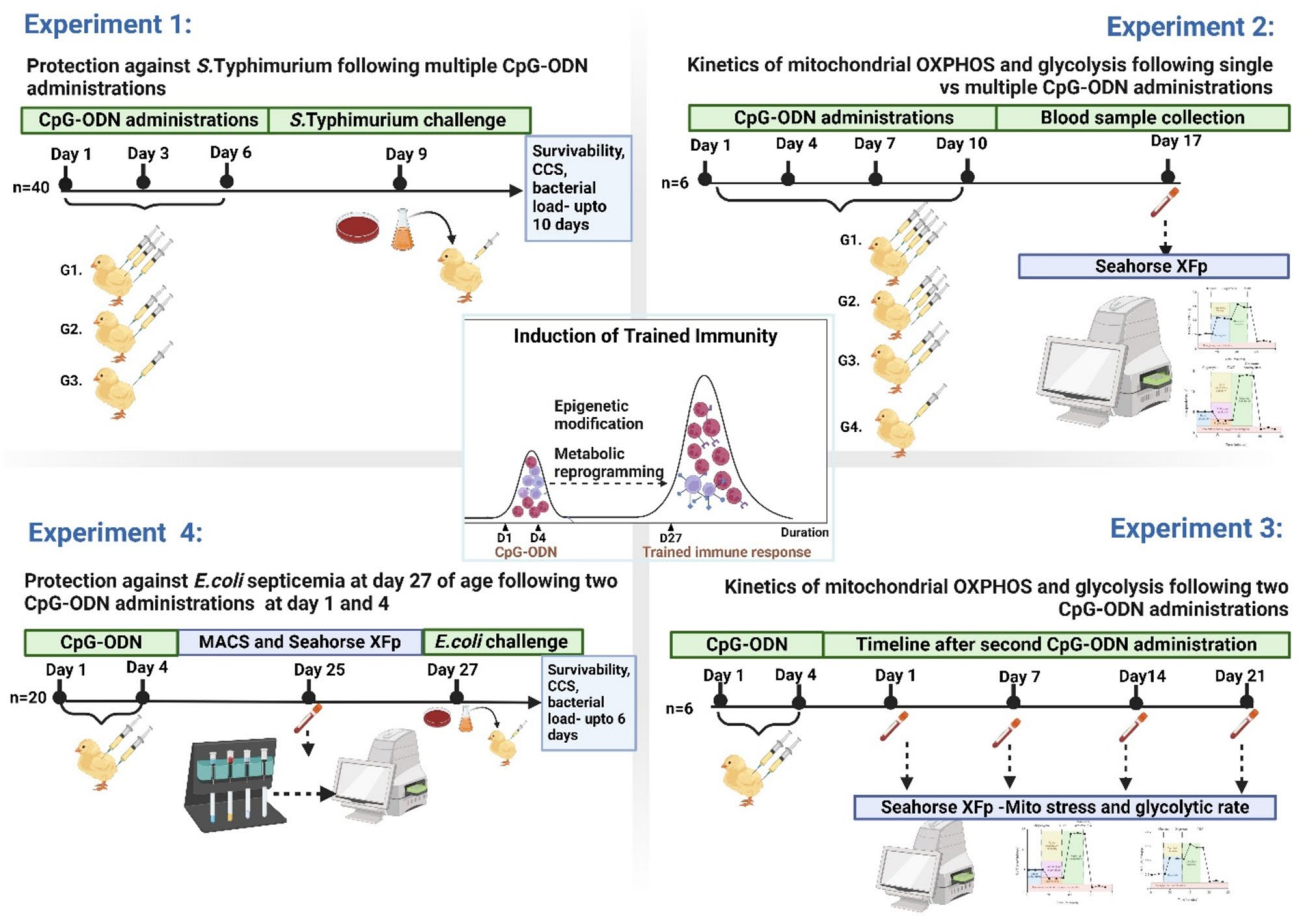
Schematic representation of the experimental design portraying the timeline of multiple CpG-ODN administrations, bacterial challenges, and sample collection for each experiment. All the experiments included a saline control group (G), (CCS = Cumulative clinical score, MACS = Magnetic activated cell sorting). Image “created with www.BioRender.com” accessed on June 8, 2024.

Schematic representation of the experimental design, timelines, and procedures undertaken in this study (Fig. 1).

### Immunoprotective efficacy of single versus multiple administration of CpG-ODN against *S. Typhimurium* septicemia

**Figure 2:**
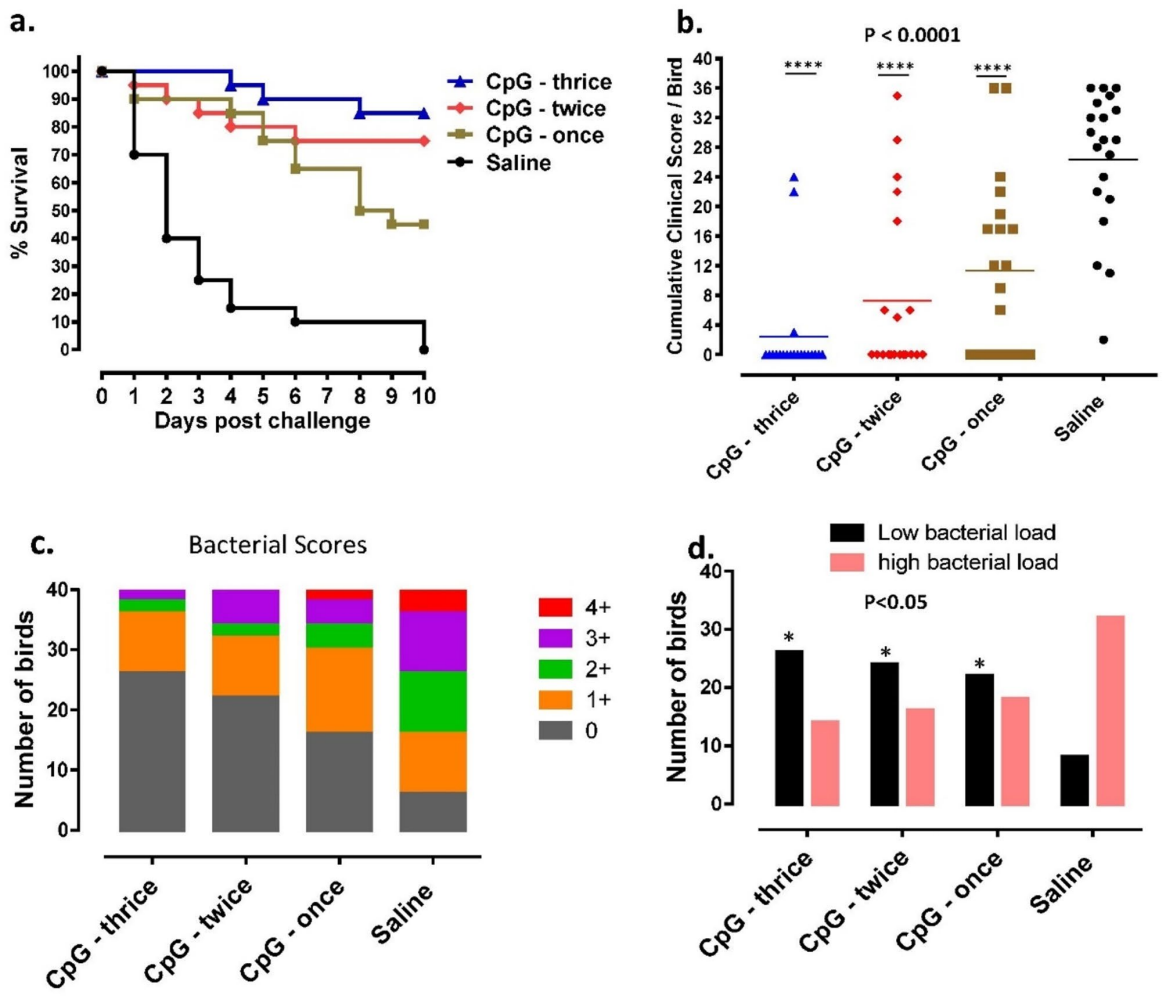
Immunoprotective efficacy of single versus multiple administrations of CpG-ODN against *S*. Typhimurium septicemia. (**a**) The survival pattern of birds over 10 d post-*S*. Typhimurium challenge (n = 40 birds/group). All CpG-ODN injected groups exhibited significantly increased (P < 0.0001) survival rates compared to the saline-injected group. Among the CpG-ODN injected groups; the group that received CpG-ODN thrice showed significantly higher (P = 0.0096) survival than the group that received CpG-ODN once. (**b**) The CCS of individual birds following *S*. Typhimurium challenge. CCS significantly decreased (P < 0.0001) in birds that received CpG-ODN compared to the group that received saline. (**c**) Bacterial load (0, few 1 +, 2 +, 3 +, & 4 +) in the air sacs of individual birds. Bacterial load was low in groups that received CpG-ODN compared to the group that received saline. (**d**) Bacterial load in the air sacs was low (no growth & few colonies) and high (scores 1 +, 2 +, 3 +, & 4 +); Birds that received CpG-ODN had significantly lower [(P < 0.0001) (χ^2^ dif = 15.68)] bacterial load compared to the saline control group.

Neonatal broiler chickens that received CpG-ODN (*i.e.* once, twice or three times) by the IM route and challenged with lethal doses of a pathogenic *S*. Typhimurium were significantly protected (P < 0.0001) compared to the saline group. When survival patterns were compared, administration of CpG-ODN three times induced a significantly higher (P = 0.0096) survival rate compared to the birds that received CpG-ODN once but there was no significant difference (P = 0.3787) between birds which received CpG-ODN twice or thrice (Fig. 2a). Birds that received CpG-ODN had significantly lower (P < 0.0001) cumulative clinical scores (CCS) following *S*. Typhimurium challenge compared to the group that received saline (Fig. 2b). Moreover, birds that received CpG-ODN had a statistically lower (χ^2^ = 15.68, P < 0.0001) bacterial load compared to the saline group. Birds that received CpG-ODN three times had significantly the lowest (P = 0.0008) load of bacteria in the air sacs, followed by CpG-ODN twice (P = 0.0057) compared to the saline group. Mortality post-bacterial challenge was characterized by airsacculitis, perihepatitis, pericarditis, or a combination of airsacculitis together with pericarditis or polyserositis. (Fig. 2c,d).

### Cellular glycolysis and mitochondrial OXPHOS capacity in PBMCs following intramuscular administration of single or multiple doses of CpG-ODN

**Figure 3:**
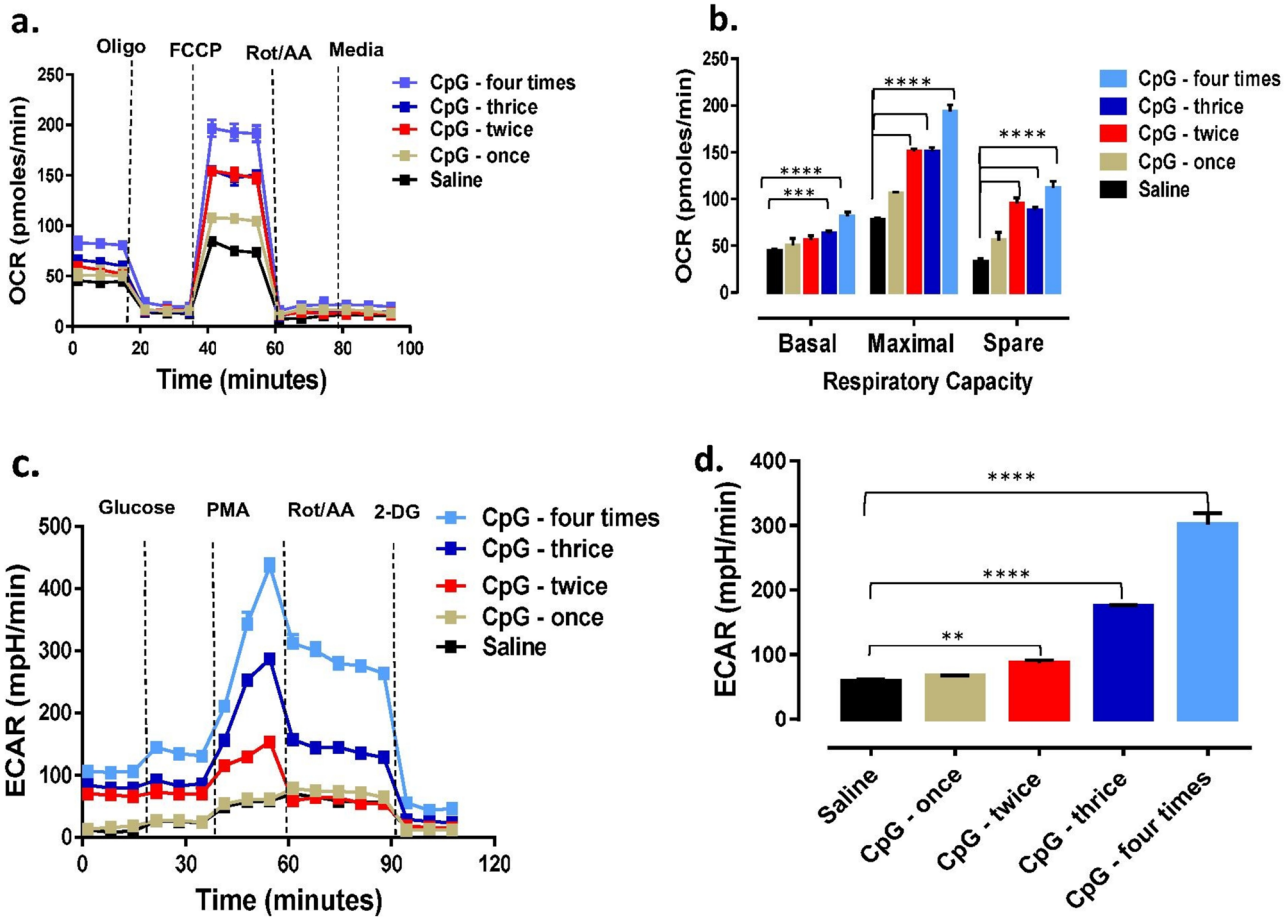
Cellular energy production capacity following single or multiple administrations of CpG-ODN. Mitochondrial respiration as oxygen consumption rate (OCR) and cellular glycolysis as extracellular acidification rate (ECAR) were measured in PBMC 7 d following the last CpG-ODN administration (n = 6 birds/group from three independent experiments/replicates). (**a**) Mitochondrial respiration or OCR changing patterns over time. OCR of the CpG-ODN group exhibited an increased number of CpG-ODN administrations compared to the group that received saline. (**b**) Statistical analysis of basal, maximal, and spare respiratory capacities of CpG-ODN and saline groups. The group that received CpG-ODN four times had significantly higher basal (P < 0.0001), maximal (P < 0.0001) and spare respiration (P < 0.0001); the group received CpG-ODN thrice had significantly higher basal (P < 0.001), maximal (P < 0.0001) spare respiration (P < 0.0001); the group that received CpG-ODN twice had significantly higher maximal (P < 0.0001) and spare respiration (P < 0.0001) than the saline control. (**c**) Cellular glycolysis or ECAR increased with the elevated number of CpG-ODN administrations compared to the group that received saline. (**d**) Significantly increased (P < 0.0001) compensatory glycolysis in groups that received CpG-ODN twice, thrice or four times than the group that received saline. (Oligo = Oligomycin, FCCP = Carbonyl cyanide-4 (trifluoromethoxy) phenylhydrazone, Rot/AA = Rotenone and Antimycin A, 2-DG = 2-Deoxy-d-glucose, PMA = Phorbol 12-myristate 13-acetate).

This experiment was conducted to investigate the effects of single versus multiple administrations of CpG-ODN on mitochondrial OXPHOS and cellular glycolysis in PBMCs 7d post-last CpG-ODN administration (Fig. 3). The groups that received CpG-ODN (*i.e.* once, twice, thrice, or four times) showed higher oxygen consumption rate (OCR) values at basal and maximal respiratory levels than the saline group (Fig. 3a). Birds which received CpG-ODN four times or three times had significantly higher (P < 0.0001 or P < 0.001) basal, maximal, and spare respiration compared to the saline control group. Birds that received CpG-ODN twice had significantly higher (p < 0.0001) maximal and spare respiration compared to the group which received saline (Fig. 3b). Cellular glycolysis, and extracellular acidification rate (ECAR) was higher in the groups that received CpG-ODN twice, thrice or four times compared to the group that received saline. Compensatory glycolysis was significantly higher in the groups treated with CpG-ODN four times (P < 0.0001), thrice (P < 0.0001) and twice (P < 0.01) compared to the group that received saline (Fig. 3c,d).

### Kinetics of cellular glycolysis and mitochondrial OXPHOS capacity in PBMCs following intramuscular administration of CpG-ODN twice over 21 days

**Figure 4:**
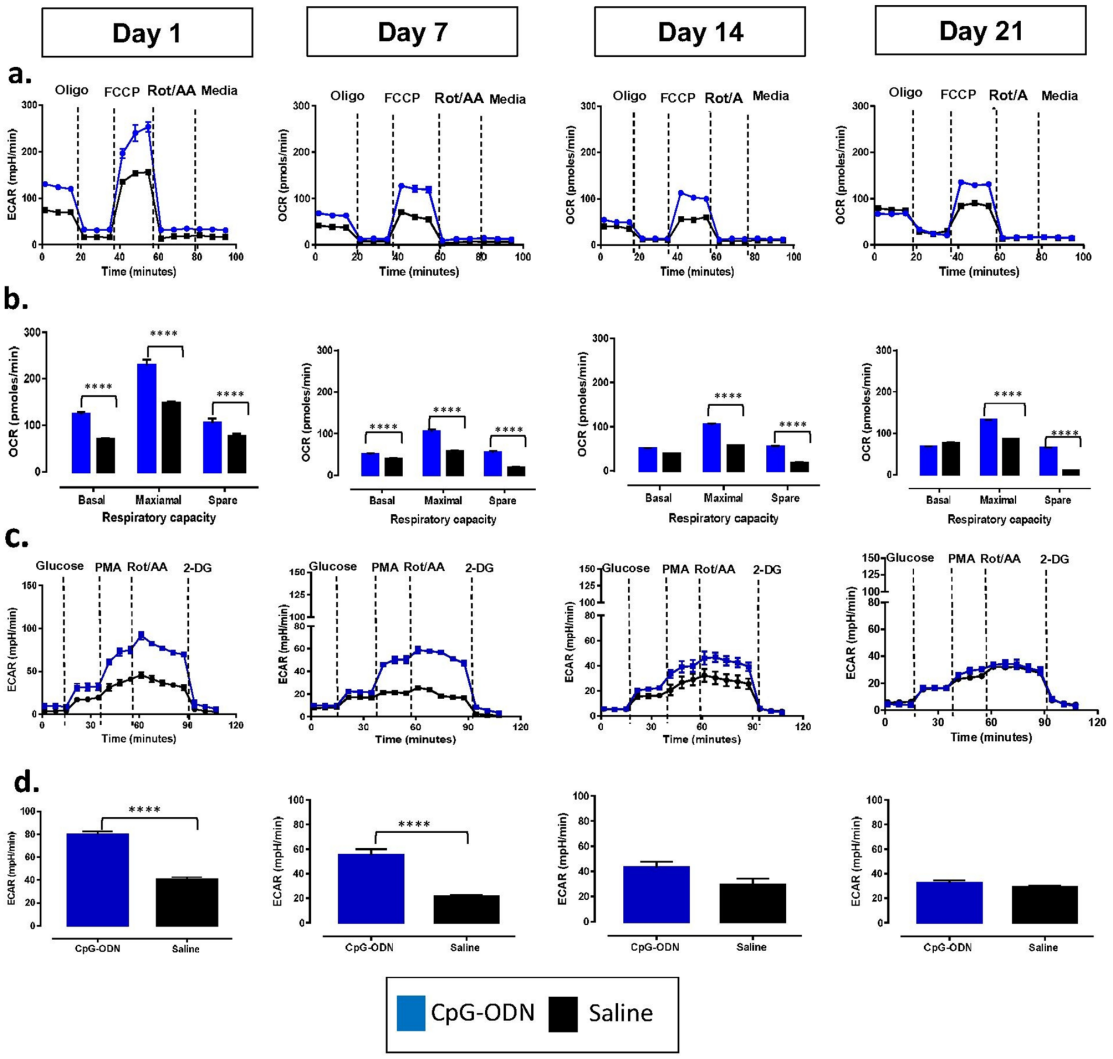
Kinetics of mitochondrial OXPHOS and glycolysis over 21 days following two CpG-ODN administrations. Metabolic outputs for oxygen consumption rate (OCR) and extracellular acidification rate (ECAR) of PBMC isolated from birds were measured at four time points; 1, 7, 14 and 21 d following the second CpG-ODN intramuscular administration (n = 6 birds/group from two independent experiments/replicates). (**a**) OCR changing pattern in birds that received CpG-ODN which had increased OCR compared to the control group that received saline at all-time points. (**b**) Birds which received CpG-ODN had significantly increased basal, maximal spare respiratory capacities at 1 and 7 d. Significant elevation of maximal and spare respiration at 14 d (P < 0.0001), and 21 d (P < 0.0001) compared to the control group. (**c**,**d**) Cellular glycolytic rates. Birds that received CpG-ODN had significantly increased ECAR at 1 and 7 d (P < 0.0001) and decreased by 14 and 21 d (non-significant, P = 0.0629 and P = 0.2278) compared to the saline control group. (Oligo = Oligomycin, FCCP = Carbonyl cyanide-4 (trifluoromethoxy) phenylhydrazone, Rot/AA = Rotenone and Antimycin A, 2-DG = 2-Deoxy-d-glucose, PMA = Phorbol 12-myristate 13-acetate).

To measure the kinetics of cellular glycolysis and mitochondrial OXPHOS, CpG-ODN was administered to birds at 1 and 4 d of age by the IM route. Peripheral blood was collected from the same group of birds at 1, 7, 14 and 21 d following the second CpG-ODN administration in this longitudinal study to measure OCR and ECAR (Fig. 4). Maximal and spare respiratory capacities were significantly increased at all-time points, at 1 d (P < 0.0001), 7 d (P < 0.0001), 14 d (P < 0.0001), and 21 d (P < 0.0001) compared to the saline group following the second administration of CpG-ODN. However, basal respiratory capacity was significantly increased only at 1 d (P < 0.0001) and 7 d (P < 0.0001), following CpG-ODN administration compared to the saline group (Fig. 4a,b). In contrast, compensatory glycolysis of PBMCs was significantly higher (P < 0.0001) in birds administered with CpG-ODN only at 1 and 7 d following the second CpG-ODN administration compared to the saline group. The differences in compensatory glycolysis between the CpG-ODN and saline groups were not significant, at 14 d (P = 0.0629) and 21 d (P = 0.2278) following the second CpG-ODN administration (Fig. 4c,d).

### Immunoprotective effects of administration of CpG-ODN twice in neonatal chickens against *E. coli* septicemia on 27 days of age

**Figure 5:**
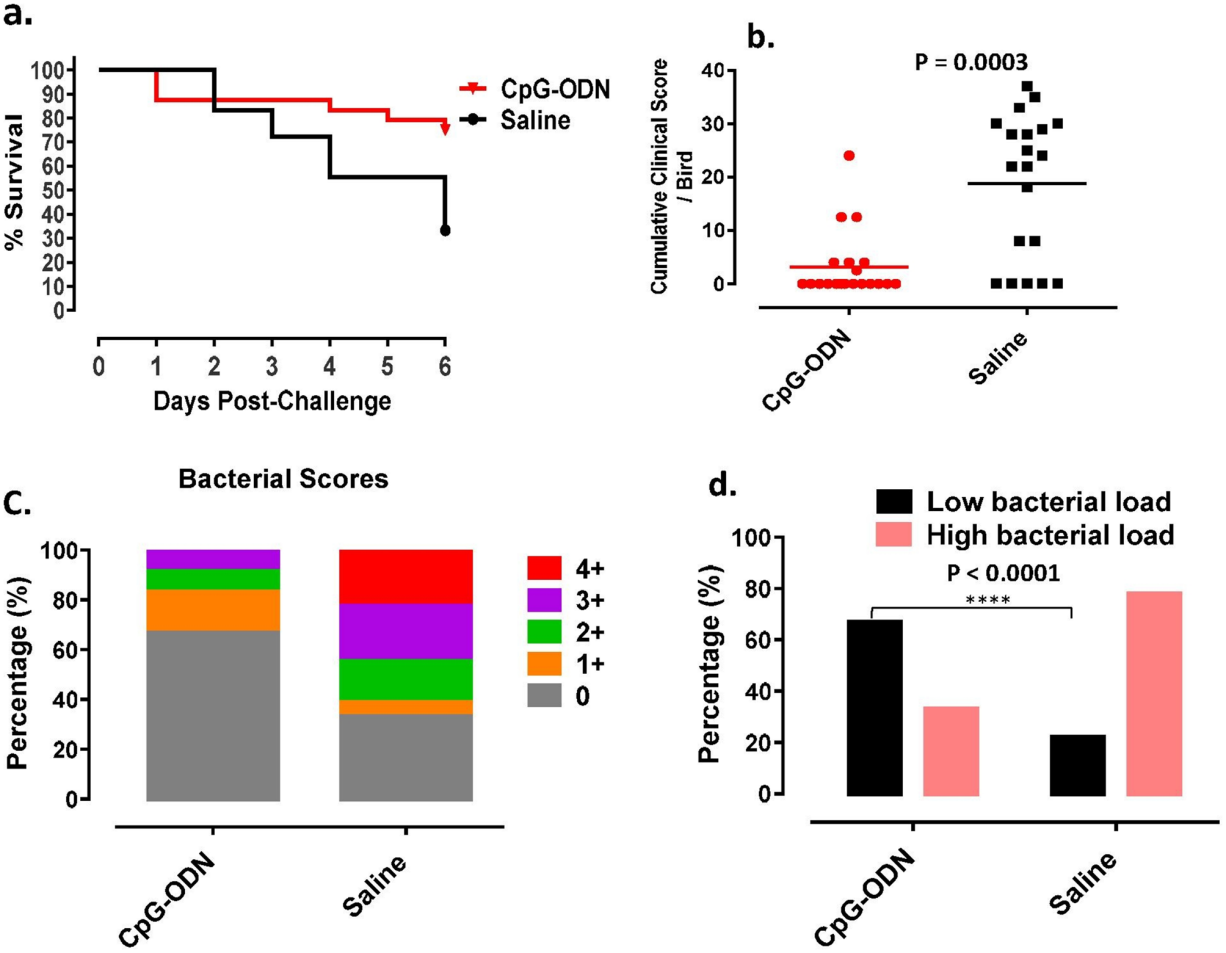
Immunoprotective effects following two administrations of CpG-ODN in birds against *E. coli* septicemia. CpG-ODN was administered in birds at 1 and 4 d of age. Birds were challenged with a lethal dose of *E. coli* at 27 d (23 d following CpG-ODN administration) (n = 20 birds/group). (**a**) The daily mortality rate of birds over 6 d following *E. coli* challenge (P = 0.0132). (**b**) Cumulative clinical score (CCS) of individual birds following *E. coli* challenge (P = 0.0003). (**c**) Bacterial load (0, few 1 +, 2 +, 3 +, & 4 +) in the air sacs. Increased bacterial loads were found in birds in the group that received saline compared to the group administered with CpG-ODN. (**d**) Birds with low bacterial loads (no growth & few colonies) and high bacterial loads (1 +, 2 +, 3 +, & 4 +). The CpG-ODN administered group had significantly [(P < 0.0001) (χ^2^ = 41.0)] lower bacterial load compared to the saline control group.

This study was conducted to investigate the immunoprotective ability of CpG-ODN which was administered twice at 1 and 4 d of age in chickens against *E. coli* septicemia after challenge at 27 d of age (Fig. 5). The survival of birds following *E. coli* challenge at 27 d of age was significantly higher (P = 0.0132) higher in the group that received CpG-ODN twice compared to the group of birds that received saline (Fig. 5a). The CCS in birds that received CpG-ODN twice was significantly lower (P = 0.0003) compared to the saline group (Fig. 5b). Birds that received CpG-ODN twice had a significantly lower (P < 0.0001) (χ^2^ = 41.0) lower bacterial load in the air sacs compared to the group of birds that received saline (Fig. 5c,d).

### Mitochondrial OXPHOS capacity in neonatal chickens 21 days following administration of CpG-ODN twice in equal number of monocytes, B cells, and T cells

**Figure 6:**
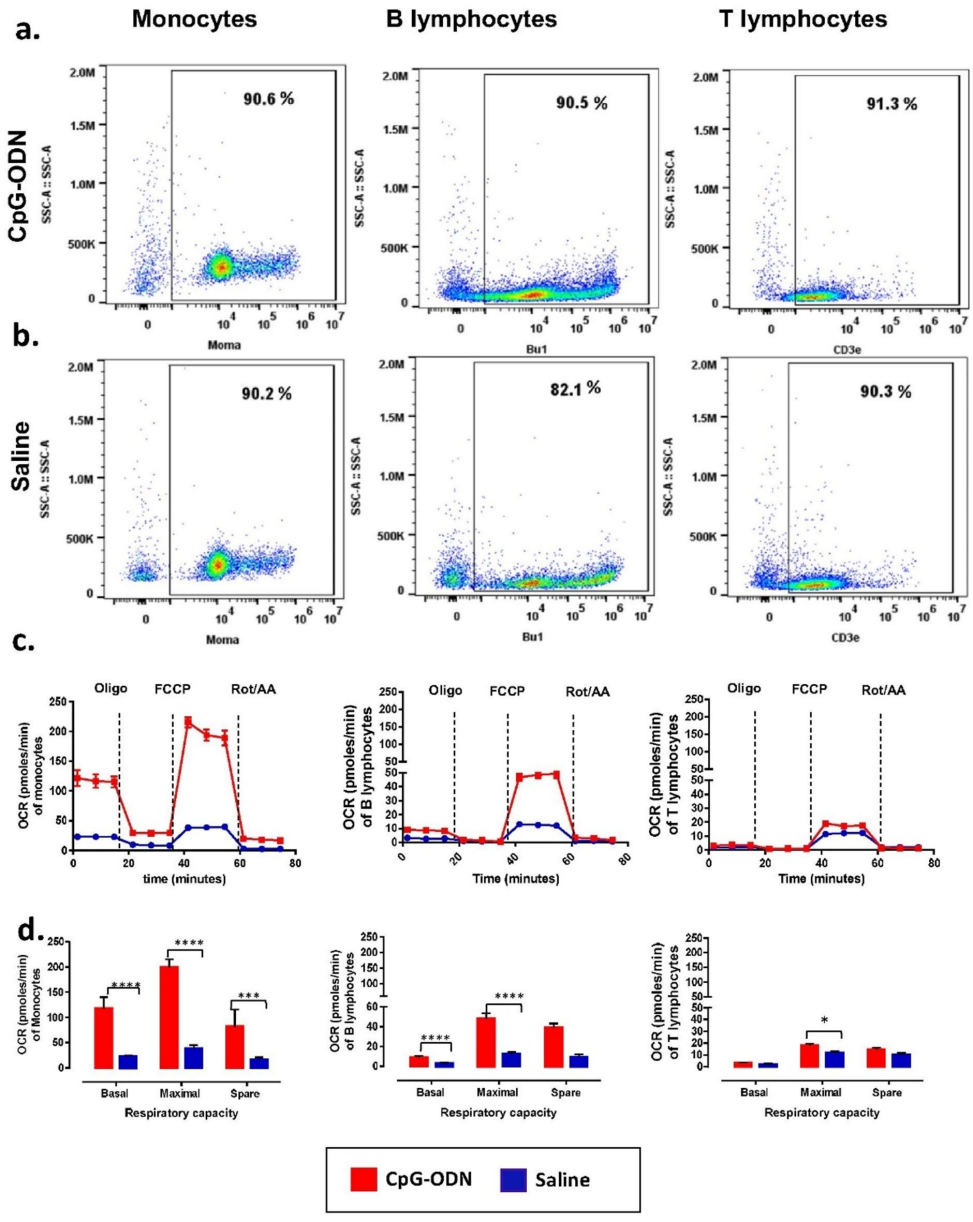
Mitochondrial energy production capacity of chickens after 21 days following 2 CpG-ODN administrations. An equal number of monocytes, B cells, and T cells were measured (n = 4 birds/group). (**a**) Monocytes, B cells, and T cells isolated from birds administered with CpG-ODN; (**b**) Monocytes, B cells, and T cells isolated from birds administered with saline. (**c**) Measurement of OCR in monocytes, B cells, and T cells. (**d**) OCR values were significantly higher in monocytes; [basal (P < 0.0001), maximal (P < 0.0001) and spare respiratory capacity (P = 0.0092)]; B cells; [maximal and spare respiratory capacity (P < 0.0001)] and T cells; [maximal (P = 0.0161)] than the saline control. (Oligo = Oligomycin, FCCP = Carbonyl cyanide-4 (trifluoromethoxy) phenylhydrazone, Rot/AA = Rotenone and Antimycin A).

The purities of monocytes, B and T cells, sorted using magnetically activated cell sorting (MACS) were more than 90% in both the CpG-ODN administered and saline groups (Fig. 6a,b). OCR values of monocytes in birds administered with CpG-ODN twice were significantly higher [basal respiration (P < 0.0001), maximal respiration (P < 0.0001), and spare respiratory capacity (P = 0.0092)] compared to the monocytes isolated from birds administered with saline. OCR values of B cells in birds administered with CpG-ODN twice were significantly higher [maximal respiration (P < 0.0001) and spare respiratory capacity (P < 0.0001)] and T cells [maximal respiration (P = 0.0.0161)] than to B cells and T cells isolated from birds administered with saline (Fig. 6c,d).

## Discussion

CpG-ODN mediated immunoprotection has been demonstrated in chickens following administration of a single prophylactic dose to induce protection against several bacterial pathogens, namely *S.* Typhimurium^40^, *Salmonella enteritidis*^41^, *E. coli*^10,42^ and *Campylobacter jejuni*^43^. However, CpG-ODN mediated immunoprotection lasts only for a few days following single administration of CpG-ODN^8,13^. In a previous study, the benefit of multiple administration of CpG-ODN had been shown in mice with prolonged protection. CpG-ODN injections by the IM route, bi-weekly for 4 months, enhanced the survival against *Francicella tularensis and Listeria monocytogenes* for up to 2 weeks^38^. Protection against *Staphylococcus aureus* increased up to 2 weeks following 2 consecutive IV injections (two days apart) in mice^39^. Here we provide novel information related to CpG-ODN mediated activation of trained immunity against important chicken bacterial pathogens.

In this study, we investigated immunoprotection against *S*. Typhimurium in broiler chickens following multiple administrations of CpG-ODN. Chickens were treated with CpG-ODN via the IM route once, twice or thrice then challenged with a lethal dose of *S*. Typhimurium at 9 d of age. We observed significantly higher protection against *S*. Typhimurium in all groups that were administered with CpG-ODN. There was also significant protection against *S*. Typhimurium in birds treated thrice with CpG-ODN compared to birds that received CpG-ODN once. Moreover, we observed an association of lower bacterial load in the body with increasing frequency of CpG-ODN administrations. Furthermore, we demonstrated a significant protection of chickens against a lethal challenge of *E. coli* septicemia 27 d following administration of CpG-ODN twice at 1 and 4 d of age. This study provides evidence of induction of trained immunity in chickens following repeated administration of CpG-ODN. Induction of trained immunity not only increased the survival of birds but also decreased the bacterial load and clinical outcome in birds following challenge with *E. coli*. Likewise, protection of mice against *L. monocytogenes* and *F. tularensis* was observed for up to 2 weeks following multiple administrations of CpG-ODN bi-weekly for 4 months^38^. Another study in mice also demonstrated protection of mice against *S*. *aureus* for 2 weeks following administration of CpG-ODN by the intravenous (IV) route twice^39^. We did not observe any adverse reactions following the administration of CpG-ODN twice, all birds were healthy during the entire study period. Here, we provide evidence of induction of trained immunity or innate immune memory in chickens following administration of CpG-ODN twice to protect them against lethal *E. coli* infection. To our knowledge, the present study is the first to describe the induction of innate immune memory or trained immunity following administration of CpG-ODN in chickens or in any other avian species. Trained immunity or innate immune memory is a unique feature of the innate immune system that enables resistance to unrelated pathogens. Innate immune cells undergo modifications and associated transcription through epigenetic rewiring leading to changes in the physiology of cells without permanent genetic changes^30^. Several studies in mice^37,44–46^, humans^33,47–49^, plants^50,51^, and invertebrates^52,53^ provide evidence of trained immunity. A study in mice demonstrated that immunization of mice with BCG, the most commonly used vaccine worldwide to prevent tuberculosis, can lead to a T cell-independent protection against secondary infections such as *Candida albicans* and *Schistosoma mansoni*^54^. A study conducted in humans demonstrated protection of people against yellow fever virus infection induced by a yellow fever vaccine strain one month following BCG vaccination due to induction of epigenetic reprogramming of the PI3K/AKT/mTOR pathway and associated cytokine production (*i.e.* IL-1β and IL-6) by monocytes^55^. Moreover, in countries where people were vaccinated with BCG, reduced disease spread, severity and death were reported during the SARS‐CoV‐2 and COVID-19 pandemics due to trained immunity^56–58^. These studies demonstrate induction of trained innate immunity following BCG vaccination and protection against viral infections. Protection of mice against *E. coli* infection following injection of zymosan twice 3–7 days prior to intraperitoneal (IP) *E. coli* challenge has also been demonstrated. This protection of mice against *E. coli* was associated with reduced bacterial load, decreased *E. coli* dissemination, improved survival, elevated cytokine production (IL-6 and TNF) and significantly elevated polymorph nuclear cells and macrophages in the peritoneal cavity. Trained immunity mediated by zymosan also protected mice against systemic *S. aureus* and *L. monocytogenes* infections^59^.

Metabolomics has become a tool for in-depth understanding of immunometabolism at the cellular level about metabolic reprogramming and innate immune memory development. Trained immunity has been described in mammalian species primarily in myeloid cells such as monocytes/macrophages, lymphoid cells such as natural killer cells, and dendritic cells^33,48,60–63^. Immune cells such as monocytes, T cells and B cells undergo changes in metabolism when cells go through stimulation, threat, or pathogen invasion to accelerate cell signaling, cytokine production and cell division^64–67^. We previously reported that administration of CpG-ODN once can induce antimicrobial immunity and changes in serum metabolite profile against *E.coli* septicemia in chickens^68^. Metabolic rewiring occurs during cell differentiation and is directed to trained immunity and a characteristic trait of trained immunity^30^. Here, we further investigated the metabolic outputs of mitochondrial OXPHOS and cellular glycolysis of immune cells after multiple administrations of (*i.e.* once, twice, thrice or four times) of CpG-ODN. Our results demonstrated significantly higher OCR and ECAR or increased mitochondrial OXPHOS and cellular glycolysis following multiple administrations of CpG-ODN compared to the saline control. This provides evidence that CpG-ODN-mediated immunoprotection in chickens is correlated with metabolic changes in immune cells. We observed a significantly high sustained mitochondrial respiration (*i.e.* basal, maximal, and spare respiratory capacity) of PBMC for 21 d following CpG-ODN administration. In contrast, cellular glycolysis was transiently increased until 7 d post-CpG-ODN administration and then decreased by 21 d following CpG-ODN administration. These results indicate that immune cells are shifting energy use from glycolysis to mitochondrial respiration. This is likely associated with immune cells transitioning to trained immune cells or innate memory cells to increase the ability to survive and maintain energy production. Our study demonstrated how major energy-producing pathways change over time as a vital process of cellular metabolic reprogramming after multiple CpG-ODN administrations. A significant increase of cellular glycolysis and mitochondrial OXPHOS was observed at early stages (*i.e.* 1 and 7 d following CpG-ODN administration) indicates that both pathways may be important in initiating trained immunity in chickens.

We quantified mitochondrial OXPHOS and glycolysis in monocytes, B cells and T cells after 21 d of CpG-ODN administration and found that mitochondrial OXPHOS in the CpG-ODN group was significantly higher in monocytes, B cells, and T cells compared to the saline control group. Furthermore, OCR values were quantitatively higher in monocytes (*i.e.* basal, maximal and spare respiratory capacities) compared to B cells and T cells. However, this observation needs be further investigated regarding the OXPHOS activity of individual immune cell subpopulations. It is possible that monocytes might play an important role in trained immunity compared to lymphocytes in chickens following CpG-ODN administration. It is noteworthy to mention that the immune cells of avian species are quite different in terms of morphology and function compared to their mammalian counterpart^69,70^. Monocytes are important innate immune cells that can provide valuable insights about the mechanisms of trained immunity. Studies indicate that in vitro training of mouse primary monocytes with β-glucan for 24 h has the potential to instruct monocytes, resulting in alterations in histone trimethylation, which suggests the involvement of epigenetic mechanisms^60^*.* One of the challenges we encountered while looking into CpG-ODN mediated trained immunity in broiler chickens is the scarcity of immune cell markers. To address this limitation, forthcoming investigations leveraging single-cell transcriptomic analysis, metabolic pathway exploration, and investigation of epigenetic alterations in immune cells after CpG-ODN administration will offer profound insights into the mechanistic pathways and cell populations underpinning trained immunity in chickens.

Bacterial infections such as *E. coli, Salmonella,* and enterococci are major infectious diseases in neonatal broiler chickens^71,72^. Furthermore, these neonatal infections lead to subsequent chronic infections, poor growth, poor feed conversion and downgrading of poultry products and increased condemnations at processing^73,74^ with associated significant economic losses and public health risks. Lack of effective vaccines against these common bacterial infections in the broiler chicken industry also leads to the increased use of antibiotics and subsequent emergence of antimicrobial resistant bacteria. Developing alternative methods to provide broad-spectrum protection for chickens against pathogens, such as exploring molecules capable of inducing trained immunity in chickens, as demonstrated in this study, is imperative. Doing so not only ensures the welfare of animals but also contributes to increased food production and enhances public health safety.

In conclusion, we have demonstrated that delivering CpG-ODN twice in neonatal broiler chickens can induce innate memory or trained immunity and protect them against lethal bacterial infections later in life. We have also demonstrated protection of neonatal broiler chickens against *S*. Typhimurium infection by administration of CpG-ODN once and multiple times at a significant level, but administration of CpG-ODN multiple times provided significant protection compared to administration of CpG-ODN once. Furthermore, our results revealed that induction of trained immunity was associated with shifting of the metabolic pathways of immune cells towards mitochondrial OXPHOS following administration of CpG-ODN twice. Shifting to mitochondrial respiration compared to energy use from glycolysis is likely associated with immune cells transitioning to trained immune cells or innate memory cells to increase the ability to survive and maintain energy production. However, it would be important to explore epigenetic modifications such as DNA methylation and transcription of long non-coding RNAs associated with trained immunity in chickens to further elucidate mechanisms.

## Materials and methods

### Housing and maintenance of experimental chickens

This work was carried out in compliance with the ARRIVE (Animal Research: Reporting of in Vivo Experiments) guidelines. This animal study was approved by the Animal Research Ethics Board, University of Saskatchewan (protocol number 20070008). All the methods were performed accordance with the guidelines and regulations of Canadian Council on Animal Care. Euthanasia was performed by cervical dislocation following the AVMA guidelines for the euthanasia of animals. Broiler chickens (Ross 308) at the day of hatch (1 d), were obtained from a commercial hatchery in Saskatchewan. Groups of broiler chicks were maintained at the Animal Care Unit, Western College of Veterinary Medicine, University of Saskatchewan, Saskatchewan, Canada. Water and an antibiotic-free commercial broiler starter ration were supplied ad libitum. Air from each room was exhausted through a HEPA filter and non-recirculated intake air circulation was supplied at 15–20 air changes/hour rate. Furthermore, air pressure differentials and strict sanitation were maintained in this isolation facility. Birds were raised at 32 °C for the first 7 days of life, after that, temperature conditions were reduced by 0.5 °C per day until a temperature of 20 °C. Lighting (30–40 lx) was provided continuously until 2 d post-hatch, thereafter lux and duration were decreased until 10–20 lx and 7 h of darkness were achieved.

### Synthetic CpG-ODN

The sequence of Class B CpG-ODN^2007^ used in all animal experiments was 5ʹ-TCGTCGTTGTCGTTTTGTCGTT-3ʹ (free of endotoxin) as previously described^14,17^. CpG-ODN was synthesized with a modified phosphorothioate backbone (Operon Biotechnologies, Inc., Huntsville, AL, USA). CpG**-**ODN was diluted in sterile pyrogen-free phosphate-buffered saline (PBS). Each bird received 50 µg of CpG-ODN in a total volume of 100 µL, via the IM route, with a 23 gauge, 1 inch needle. The control group was injected PBS via the IM route.

### Bacterial challenge preparation

#### *Salmonella* Typhimurium

*S*. Typhimurium was isolated from a 25-week-old broiler breeder chicken with septicemia and *S.* Typhimurium culture was prepared for *c*hallenge studies as previously described^40^. Briefly, *S.* Typhimurium was cultured on tryptic soy agar containing 5% sheep blood (Thermo Scientific, Canada), and incubated aerobically for 18–24 h at 37 °C. Then, 2–3 colonies were added into 200 mL of Luria broth (Miller, BDH, Poole, United Kingdom) in a 500 mL Erlenmeyer flask and incubated at 37 °C for 16–18 h on a shaker with shaking rate of 200 rpm. After incubation, the cultures contained approximately 1 × 10^9^ colony-forming units (CFU) of stationary-phase of bacteria. The *S.* Typhimurium inoculum was serially diluted with saline to achieve 1 × 10^7^ CFU/mL and 1 × 10^8^ CFU/mL in a total volume of 250 µL. Mortality, CCS, and bacterial load in the air sac were recorded over 10 d following the challenge.

#### *E. coli*

The *E. coli* strain was isolated from a turkey with septicemia; the bacteria belongs to serogroup O2 isolate and is nonhemolytic, serum-resistant, produced aerobactin, contained K1 capsule and Type 1 pili. The *E. coli* challenge preparation was conducted as previously described^8,17^. Briefly, *E. coli* was cultured for 18–24 h at 37 °C on 5% Columbia sheep blood agar (Becton, Dickinson and Company, Maryland, USA). A single colony of bacteria from the agar plate was added to 100 mL of Luria broth in a 250 mL Erlenmeyer flask. The culture was grown at 37 °C for 16–18 h, shaking at 150 rpm. After incubation, the cultures contained approximately 1 × 10^9^ CFU/mL of stationary phase of bacteria. These were further diluted in sterile saline to the concentration of bacteria required in the challenge experiments. The *E. coli* challenge was conducted with 1 × 10^6^ CFU/mL and 1 × 10^7^ CFU/mL of bacteria in a total volume of 250 µL of saline, subcutaneously in the neck^68,75^. Mortality, CCS, and bacterial load in the air sac were recorded over 6 d post-challenge.

### Seahorse extracellular flux analysis (XFp) assay using peripheral blood mononuclear cells (PBMCs)

Real-time and live cell analysis of glycolysis based on extracellular acidification rate (ECAR) and mitochondrial oxidative phosphorylation (OXPHOS) based on the oxygen consumption rate (OCR) were quantified using the Seahorse XFp Analyzer, https://www.agilent.com/en/product/cell-analysis/real-time-cell-metabolic-analysis/xfsoftware/ seahorse-xfp-analyzer-software-740905 (Agilent Technologies, Santa Clara, CA, USA)^76,77^.

First, the XFp culture plates were coated with 50 μL poly-D lysine (Sigma-Aldrich, Canada) in distilled water (1:8) and incubated at 4 °C for 24 h and sensor cartridges were hydrated overnight with 200 μL XF calibrant fluid (Agilent Technologies, Canada) in wells and 400 μL in each moat at 37 °C without CO_2_. XF calibrant fluid and Roswell Park Memorial Institute (RPMI) culture media (Agilent Technologies, Canada) were incubated overnight at 37 °C without CO_2_. At the time of the experiment, peripheral blood samples were collected into sodium heparin tubes from the brachial vein using a 22-gauge, 1-inch hypodermic needle. Heparinized blood was mixed with the same volume of PBS (pH = 7.4), layered on 3 mL Histopaque 1077® (Sigma Aldrich, Oakville, ON, Canada), and centrifuged for 30 min at 400 × g at 20 °C to collect the PBMC layer. The PBMC layer was washed with 5 mL pre-warmed RPMI and spun at 300 × g for 5 min at 20 °C twice. The PBMC pellet was then re-suspended in 2–3 mL of RPMI and the live cells were counted using trypan blue and added 1.5 × 10^5^ cells per well. After spinning for 2 min at 300 × g at 20 °C, 130 μL of RPMI was added to each well (total volume 180 µL/well) and the plate was incubated at 37 °C without CO_2_ for 60 min. A hydrated sensor cartridge was then loaded in the Seahorse XFp Flux Analyzer with stimulants and inhibitors for cell mitochondrial stress assay and glycolytic rate assay.

### Mitochondrial stress-assay

Seahorse XFp cell culture media was prepared using RPMI 1640 XF Medium pH 7.4 (with 1 mM HEPES, without phenol red, glucose, pyruvate, and L-glutamine), 1 mM pyruvate (Agilent Technologies Canada ), 2 mM L-Glutamine (Agilent Technologies Canada), and 10 mM glucose (Seahorse Agilent, Santa Clara, CA, USA). Four ports of the XFp cartridge were loaded with different reagents. (a) 20 µL of oligomycin (1.5 µM/well) was added into port A, which inhibits mitochondrial OXPHOS based on the OCR (ATP synthase inhibitor) to shift cellular energy production toward glycolysis (*i.e*., ECAR values increase), (b) 22 µL of carbonyl cyanide p-(tri-fluromethoxy) phenyl-hydrazone (FCCP) (2.5 µM/well) was added to port B to depolarize the mitochondrial membrane to increase in oxygen consumption (*i.e.*, OCR values increase, cells achieve maximal ECAR), (c) 25 µL of rotenone/antimycin A (Rot/AA) mixture (0.5 µM/well) was added in the port C to inhibit complex 1 and 3 of electron transport chain of the mitochondrial respiration, and (d) Port D was loaded with culture media.

### Glycolytic rate assay

The assay procedure was similar to mitochondrial stress assay, except the cell culture media was prepared without glucose (only RPMI 1640 XF medium + pyruvate (1 mM) + (2 mM) l-Glutamine solution): (a) 20 µL of glucose (10 mM/well) was added to port A to stimulate aerobic glycolysis; (b) 22 µL of phorbol 12-myristate 13-acetate (PMA) (2.5 ng/mL/well) was added specifically to activate protein kinase C (PKC); nuclear factor-kappa B (NF-κB) was added to port B to reach the highest glycolytic capacity (ECAR); (c) 25 µL of Rot/AA (0.5 µM/well) was added to port C, and (d) 27 µL of 2-deoxy-d-glucose (2-DG) (50 mM/well) was added into port D to competitively inhibit cellular glycolysis.

### Magnetic activated cell sorting (MACS) for mitochondrial stress assay

Blood samples (3–4 mL/bird) were collected into heparinized tubes from the brachial vein using a 22-gauge, 1-inch hypodermic needle. Blood samples were mixed with the same volume (1:1) of 4 mL of PBS with 1% penicillin–streptomycin (Thermo Fisher Scientific Inc. Waltham, USA) and layered on 3 mL histopaque 1077® and centrifuged for 20 min at 560×*g* at 20 °C to collect the PBMC layer. The PBMC layer was collected in a new 15 mL centrifuge tube and washed three times with MACS buffer prepared using PBS supplemented with 0.5% bovine serum albumin (Sigma Aldrich, Oakville, ON, Canada) and 2 mM disodium ethylenediaminetetraacetate dehydrate (Sigma Aldrich, Oakville, ON, Canada). Live cells were counted using trypan blue. Monocytes and B cells at the concentration of 1 × 10^7^ and T cells at the concentration of 5 × 10^7^ were re-suspended in MACS buffer. MACS was performed according to the manufacturer’s instructions (Miltenyl Biotec Inc., San Diego, CA). Monocytes, B cells, and T cells, were stained with mouse anti-chicken monocyte/macrophage-PE (KUL01), mouse anti-chicken Bu-1-PE, and mouse anti-chicken CD3-PE (CT-3) (Southern Biotech, Birmingham, USA), respectively, and incubated for 20 min on ice in the dark. Then, cells were washed twice with 10 mL of MACS buffer and centrifuged at 300×*g* at 4 ℃ for 10 min. After washing, the cell pellet was re-suspended in the MACS buffer. Anti-PE microbeads (Miltenyi Biotec, Auburn, CA, USA) were added into tubes and incubated for 15 min on ice in the dark. The washing step was repeated with 10 mL of MACS buffer and cells were re-suspended in MACS buffer. OctoMACS™ separator and MS columns (Miltenyi Biotec, Auburn, CA, USA) were used to sort monocytes, B cells and T cells. The number and viability of sorted cells after sorting were determined using a hemacytometer and a trypan blue exclusion method. Live and dead cells were confirmed using 7-amino-actinomycin D (7-AAD) dye, followed by incubation for 20 min at 4 °C. Cells were washed three times and re-suspended in ~ 300 µL of flow cytometric buffer (PBS containing 2% fetal bovine serum). Samples were processed for flow cytometric analysis. Cells were gated based on a forward and side scatter. Fluorescence minus one control was used to identify and gate positive populations. Flow cytometry data were acquired by CytoFLEX Flow Cytometer CytoExpert, https://beckmancoulter.csod.com/ (Beckman Coulter, Carlsbad, CA). FlowJo v10.8.1, https://flowjo.com/ (Tree Star, Ashland, OR, USA) software was used to analyze data.

### Experimental design

#### Immunoprotective efficacy of single versus multiple administration of CpG-ODN against *S. Typhimurium* septicemia

Day-old broiler chickens were randomly allocated into four groups, each group containing 40 birds (n = 40/group). CpG-ODN (50 µg/bird) was injected by the IM route in the thigh muscle; Group 1 received CpG-ODN at 1, 3 and 6 d of age; Group 2 received CpG-ODN at 3 and 6 d age; Group 3 received CpG-ODN at 6 d of age; Group 4 received saline as a negative control group. *S.* Typhimurium challenge was conducted at 9 d of age, half of the birds in each group (n = 20/group) were given the low dose (1 × 10^7^ CFU/bird) and the other half received the higher dose (1 × 10^8^ CFU/bird) as previously described^40^. Birds were monitored three times per day for 10 d post-challenge. Clinical signs and a daily CCS were assigned to each bird as previously described (36): briefly, 0 = normal; 0.5 = slightly abnormal appearance, slow to move; 1 = depressed, reluctant to move; 1.5 = reluctant to move, may take a drink and peck; 2 = unable to stand or reach for food or water; and 3 = found dead. Chicks who received a clinical score of 2 were euthanized by cervical dislocation. A CCS was given at the end of the trial, with each bird given a sum of daily clinical scores, as previously described^40^. Dead or euthanized chicks were necropsied immediately. All remaining birds were euthanized at 10 d post-challenge. Swabs were taken from the air sacs, and a semi-quantitative estimate of bacteria isolation was conducted on 5% Columbia sheep blood agar by the quadrant streaking method. Bacterial growth on these cultures was recorded from 0 to 4 + , where 0 = no growth; or few = less than 5 colonies; 1 +  = growth of bacteria on quadrant 1; 2 +  = growth of bacteria on quadrants 1 and 2; 3 +  = growth of bacteria on quadrants 1, 2 and 3; and 4 +  = growth of bacteria on all quadrants 1– 4 as reported previously^10^*.*

#### Cellular glycolysis and mitochondrial energy production capacity in PBMCs following intramuscular administration of single or multiple doses of CpG-ODN

Neonatal broiler chickens were randomly allocated into 5 groups. CpG-ODN (50 µg/bird) was injected in birds three days apart by the IM route; (1) 1, 4, 7 and 10 d of age; (2) 4, 7 and 10 d of age; (3) 7 and 10 d of age; (4) 10 d of age and; (5) control group received saline. Peripheral blood samples were collected from the brachial wing vein after 7 d following the last administration of CpG-ODN (17 d of age) for Seahorse extracellular flux analysis for both OXPHOS and cellular glycolysis (n = 6/group). Data are presented in line and bar graphs as mean + standard error of the mean (SEM).

#### Kinetics of cellular glycolysis and mitochondrial OXPHOS capacity in PBMCs following intramuscular administration of CpG-ODN twice over 21 days

Neonatal broiler chicks were randomly allocated into three groups. CpG-ODN (50 µg/bird) was injected by the IM route; (1) 1 and 4 d of age; (2) 4 d of age and; (3) control group received saline. Peripheral blood samples were collected at four time points (1, 7, 14 and 21 d) from the same birds following the last CpG-ODN administration in this longitudinal study. Peripheral blood was processed to quantify mitochondrial respiration and cellular glycolysis using Seahorse extracellular flux analysis for both OXPHOS and cellular glycolysis (n = 6/group). Data are presented in line and bar graphs as mean + SEM.

#### Immunoprotective effects of administration of CpG-ODN twice in neonatal chickens against *E. coli* septicemia on 27 days of age

Neonatal broiler chickens were administered with CpG-ODN (50 µg/bird) by the IM route at 1 and 4 d of age (n = 40/group). The control group received saline by the IM route. Birds were challenged with *E. coli* at 27 d of age (23 d after the second CpG-ODN injection) with either a low dose (1 × 10^6^ CFU/mL) or high dose (1 × 10^7^ CFU/mL) of *E. coli* by the subcutaneous route in the neck as previously described (n = 20/group)^68,75^. Birds were monitored three times per day for 7 d post-challenge. Clinical scoring and bacterial load from the air sac were measured as described above (A).

#### Mitochondrial OXPHOS capacity in neonatal chickens 21 days following administration of CpG-ODN twice in equal number of monocytes, B cells, and T cells

This experiment was conducted in groups of birds, used in the above experiment D, to measure OCR values of purified monocytes, B cells, and T cells at the concentration of 3 × 10^5^ cells per well. Peripheral blood was collected at 25 d (21 d following the second CpG-ODN administration) from the brachial wing vein in heparinized syringes and conducted OXPHOS (n = 4/group). Monocytes, B cells and T cells were sorted by MACS as described above. Purity was more than 90% of each cell type. Seahorse extracellular flux analysis was performed to quantify OCR of mitochondria. Data are presented in line and bar graphs as mean + SEM.

### Statistical analysis

Survival, CCS, bacterial load of the air sac of birds, cellular mitochondrial respiration (basal, maximal, and spare respiration), and compensatory glycolysis were analyzed using GraphPad Prism ver.6, https://www.graphpad.com/ (GraphPad Software Inc., San Diego, CA). The significance level was interpreted as P < 0.05. Log-rank test (Mantel-cox) and the chi-square statistic were used to compare survival patterns and median survival time. Cellular metabolism analysis, minimum, maximum, and spare respiratory capacity, and compensatory glycolysis were analyzed using an unpaired *t*-test (two-tailed). A non-parametric one-way ANOVA test (Kruskal–Wallis test) was used to analyze multiple administrations of CpG-ODN (*i.e.* once, twice, thrice and four times) versus saline control. Further, Dunnett's multiple comparison was used as a post hoc test following ANOVA to assess significance among each treatment group compared to the saline control group.

## Data Availability

The data used to support the findings of this study are available from the corresponding author upon request.
